# Correction: Analyzing the impact of the size of fluoro and chloro substituents on induced mesomorphism in hydrogen bonded liquid crystals

**DOI:** 10.1039/d4ra90077d

**Published:** 2024-07-15

**Authors:** M. K. Sonali, Rajeev K. Sinha, Silpa Elizabeth Peter, N. V. Anil Kumar, Nirmal Mazumder, Sindhoora Kaniyala Melanthota, Mohammed Azeezulla Nazrulla, Poornima Bhagavath

**Affiliations:** a Department of Chemistry, Manipal Institute of Technology, Manipal Academy of Higher Education Manipal 576 104 Karnataka India poornima.nayak@manipal.edu; b Department of Physics, Birla Institute of Technology Mesra Ranchi 835215 Jharkhand India; c Department of Biophysics, Manipal School of Life Sciences, Manipal Academy of Higher Education Manipal Karnataka 576104 India

## Abstract

Correction for ‘Analyzing the impact of the size of fluoro and chloro substituents on induced mesomorphism in hydrogen bonded liquid crystals' by M. K. Sonali *et al.*, *RSC Adv.*, 2024, **14**, 20398–20409, https://doi.org/10.1039/D3RA08569D

The authors regret that the names of the hydrogen bonded compounds in Tables 1–3 were not correctly given in the original article. The corrected versions of Tables 1–3 are shown herein.

**Table 1:** DSC phase transition temperature (°C) and the corresponding enthalpy (J g^−1^) of P18 with fluorobenzoic acids^a^

Hydrogen bonded compound	Method	Transition	Transition temperatures (°C) (enthalpy J g^−1^)	(Δ*T*)_LC_
P18:2FBA	DSC (h)	Cryst → SmA	72.0 (4.57)	
		SmA → Iso	89.45 (83.45)	
	DSC (c)	Iso → SmA	74.26 (1.50)	13.24
		SmA → Cryst	61.02 (68.67)	
P18:3FBA	DSC (h)	Cryst → SmA	74.06 (5.06)	
		SmA → Iso	101.41 (58.58)	
	DSC (c)	Iso → SmA	78.78 (1.72)	12.79
		SmA → Cryst^1^	65.99 (34.15)	
		Crystl → Cryst^2^	61.92 (6.04)	
P18:4FBA	DSC (h)	Cryst^l^ → Cryst^2^	73.53 (20.22)	
		Cryst^2^ → SmA	89.58 (73.03)	
		SmA → Iso	107.36 (5.93)	
	DSC (c)	Iso → SmA	103.33 (1.86)	28.02
		SmA → Cryst^1^	75.31(26.88)	
		Cryst^l^ → Cryst^2^	67.03 (64.09)	

a DSC (h) is heating cycle, DSC (c) is cooling cycle. (Δ*T*)_LC_ is the thermal range of mesomorphism obtained from the cooling cycle.

**Table 2:** DSC phase transition temperature (°C) and the corresponding enthalpy (J g^−1^) of P18 with chlorobenzoic acids

Hydrogen bonded compound	Method	Transition	Transition temperatures (°C) (enthalpy J g^−1^)	(Δ*T*)_LC_
P18:2ClBA	DSC (h)	Cryst → SmA	51.27 (13.88)	
		SmA → Iso	71.68 (84.68)	
	DSC (c)	Iso → SmA	58.38 (21.57)	9.02
		SmA → Cryst	49.36 (29.04)	
P18:3ClBA	DSC (h)	Cryst → SmA	72.75 (18.40)	
		SmA → Iso	87.95 (40.19)	
	DSC (c)	Iso → SmA	68.69 (53.48)	9.13
		SmA → Cryst	59.56 (23.20)	
P18:4ClBA	DSC (h)	Cryst → SmA	75.46 (36.78)	
		SmA → Iso	98.57(28.61)	
	DSC (c)	Iso → SmA	82.16 (29.29)	20.06
		SmA → Cryst	62.10 (52.71)	

**Table 3:** DSC phase transition temperature (°C) and the corresponding enthalpy (J g^−1^) of P8 with fluoro and chlorobenzoic acids

Hydrogen bonded compound	Method	Transition	Transition temperatures (°C) (enthalpy J g^−1^)	(Δ*T*)_LC_
P8:2FBA	DSC (h)	Cryst → Iso	66.42 (50.09)	
	DSC (c)	Iso → SmA	53.69 (1.61)	25.64
		SmA → Cryst	28.05 (26.61)	
P8:3FBA	DSC (h)	Cryst → Iso	91.52 (59.96)	
	DSC (c)	Iso → SmA	69.08 (3.34)	25.14
		SmA → Cryst	43.94 (51.45)	
P8:4FBA	DSC (h)	Cryst → SmA	61.44 (40.49)	
		SmA → Iso	a	
	DSC (c)	Iso → SmA	99.71 (6.09)	56.57
		SmA → Cryst	43.14(41.65)	
P8:3ClBA	DSC (h)	Cryst → Iso	72.00(51.88)	
	DSC (c)	Iso → SmA	66.03 (5.86)	12.84
		SmA → Cryst	53.19(48.92)	
P8:4ClBA	DSC (h)	Cryst → SmA	91.73 (35.04)	
		SmA → Iso	117.26 (6.34)	
	DSC (c)	Iso → SmA	109.57 (2.55)	52.42
		SmA → Cryst	57.15(20.28)	

a Not resolved.

The Royal Society of Chemistry apologises for these errors and any consequent inconvenience to authors and readers.

